# Hornerin, an S100 family protein, is functional in breast cells and aberrantly expressed in breast cancer

**DOI:** 10.1186/1471-2407-12-266

**Published:** 2012-06-22

**Authors:** Jodie M Fleming, Erika Ginsburg, Shannon D Oliver, Paul Goldsmith, Barbara K Vonderhaar

**Affiliations:** 1 Department of Biology, North Carolina Central University, 1801 Fayetteville Street, Mary Townes Science Complex, Rm 2247, Durham, NC 27707, USA; 2 Mammary Biology and Tumorigenesis Laboratory, Center for Cancer Research, NCI, Bethesda, MD 20892-4254, USA; 3 Antibody Production and Purification Unit, National Cancer Institute, National Institutes of Health, Bethesda, MD 20892, USA

**Keywords:** Hornerin, S100 protein, Mammary gland, Breast cancer, Apoptosis, Protein fragmentation

## Abstract

**Background:**

Recent evidence suggests an emerging role for S100 protein in breast cancer and tumor progression. These ubiquitous proteins are involved in numerous normal and pathological cell functions including inflammatory and immune responses, Ca^2+^ homeostasis, the dynamics of cytoskeleton constituents, as well as cell proliferation, differentiation, and death. Our previous proteomic analysis demonstrated the presence of hornerin, an S100 family member, in breast tissue and extracellular matrix. Hornerin has been reported in healthy skin as well as psoriatic and regenerating skin after wound healing, suggesting a role in inflammatory/immune response or proliferation. In the present study we investigated hornerin’s potential role in normal breast cells and breast cancer.

**Methods:**

The expression levels and localization of hornerin in human breast tissue, breast tumor biopsies, primary breast cells and breast cancer cell lines, as well as murine mammary tissue were measured via immunohistochemistry, western blot analysis and PCR. Antibodies were developed against the N- and C-terminus of the protein for detection of proteolytic fragments and their specific subcellular localization via fluorescent immunocytochemisty. Lastly, cells were treated with H_2_O_2_ to detect changes in hornerin expression during induction of apoptosis/necrosis.

**Results:**

Breast epithelial cells and stromal fibroblasts and macrophages express hornerin and show unique regulation of expression during distinct phases of mammary development. Furthermore, hornerin expression is decreased in invasive ductal carcinomas compared to invasive lobular carcinomas and less aggressive breast carcinoma phenotypes, and cellular expression of hornerin is altered during induction of apoptosis. Finally, we demonstrate the presence of post-translational fragments that display differential subcellular localization.

**Conclusions:**

Our data opens new possibilities for hornerin and its proteolytic fragments in the control of mammary cell function and breast cancer.

## Background

The S100 protein family, consisting of over 20 members, constitutes the largest subgroup of calcium binding proteins. These proteins share amino acid sequence similarity as well as the functional EF-hand structure motif, which plays a key role in calcium binding through a helix-loop-helix topology. Proteins containing this motif are involved in virtually all normal and pathological cell functions including gene transcription, inflammatory and immune responses, regulation of protein phosphorylation, transcription factors, anti-microbial responses, Ca^2+^ homeostasis, the dynamics of cytoskeleton constituents, as well as cell proliferation, differentiation, and death [1-3]. Given the global importance of these proteins, inhibitors of specific S100 proteins are currently being developed as therapeutics for diseases including diabetes mellitus, heart failure, neurological diseases, and several types of cancer [2,4].

The role of S100 protein in breast cancer is only beginning to emerge. A recent observational study demonstrated upregulation of S100A1, S100A2, S100A4, S100A6, S100A8, S100A9, S100A10, S100A11, and S100A14 in basal-type breast cancers compared to non-basal types. In the same study, it was determined that expression of S100A8 and S100A9 were elevated in high grade compared to low grade tumors and estrogen receptor (ER) negative tumors compared to ER positive tumors [5]. Mechanistic studies demonstrated that overexpression of S100A4 induced metastatic capability in non-metastatic breast cancer cells and stimulated metastasis of benign tumors in transgenic mouse model systems [6]. S100A7 was shown to be upregulated in high-grade ductal carcinoma *in situ,* and is correlated with poor prognosis in estrogen negative breast cancer. *In vitro*, S100A7 overexpression increased breast cancer cell growth, invasiveness, and increased tumorigenicity in a xenograft mouse model [7,8]. On the other hand, S100A2 expression was found to be reduced as breast cancer progressed from carcinoma *in situ* to carcinoma [9]. Corresponding to this observation, S100A2 has also been proposed as a tumor suppressor in early stage lung carcinogenesis [10].

We recently performed mass spectrometry analysis of the extracellular matrix of whole breast tissue with the goal of determining underlying differences in the normal breast microenvironment between premenopausal African- and Caucasian-American women [11]. Premenopausal African-American women suffer disproportionately from breast cancer mortality compared to Caucasian women. Both social and biological mechanisms are contributory, including a higher prevalence of aggressive basal-like breast cancers in African-American women. Hornerin, an S100 protein family member, was detected in significantly higher abundance in the Caucasian-American samples. Therefore we further investigated the biological functions of this protein, only to find that little is known.

Hornerin was first characterized in the mouse embryo epidermis and was also detected in the skin, tongue, and forestomach of the adult tissues examined [12]. Hornerin contains a Ca^2+^ binding EF-hand domain at the N terminus followed by a spacer sequence and an extensive repetitive domain rich in glycine and serine [12]. Its similarity in structural features, expression profile, extensive posttranslational proteolytic processing, and tissue localization to profilaggrin indicated a role in keratinocyte cornification. Additional studies demonstrated the presence of hornerin in regenerating, psoriatic and healthy human skin, and that hornerin is a component of cornified cell envelope [13-15]. While it might initially seem peculiar that a protein involved in cornification of the skin is found in breast tissue, it is important to recall the evolutionary development of the mammary gland. In all mammals, the mammary gland organogenesis arises from a localized thickening of the epidermis. An elevation of the epidermal mammary crest and the development of a milk-line on both sides of the mid-ventral line of the embryo form the mammary buds, which eventually progress to form the functional mammary gland [16]. Indeed, other proteins involved in epidermal/skin function have been shown to perform roles in mammary gland physiology. Neuregulin3 regulates the cell fate of pluripotent epidermal cells, including those that ultimately differentiate into progenitor cells of the mammary gland [17]. Additionally, LMO-4 a member of the LIM-only family of transcriptional co-regulatory proteins functions in both epidermal cell migration and mammary gland differentiation [18,19].

Herein, we demonstrate hornerin expression in human breast tissue and mammary epithelial and stromal cells, its regulation throughout postnatal mammary developmental stages in murine tissue, as well as its expression in correlation with breast cancer subtypes. Furthermore, we show that proteolytic fragments of hornerin have distinctive intracellular localization and that induction of apoptosis/necrosis upregulates hornerin expression in breast cells. Collectively, these data support a novel role for hornerin in the regulation of mammary cell function.

## Methods

### Cell culture

T47D and MDA MB231 cell lines were obtained from American Type Culture Collection (ATCC; Manassas, VA) and cultured as instructed. MCF10AI cells [20] were a kind gift of F.R. Miller, Wayne Sate University, Detroit, MI, and were maintained as recommended by ATCC. Cells were passaged using trypsinization (0.05% trypsin-EDTA; Invitrogen, Gaithersburg, MD) and counted on a hemocytometer using trypan blue exclusion.

Peripheral blood monocytes and macrophages were collected from premenopausal women undergoing apheresis. Collection of patient samples was performed in accordance with the Helsinki Declaration under the guidelines of the National Cancer Institute Review Board, protocol number 99-CC-0168. Written informed consent was obtained from all human subjects as specified in the protocol. Monocytes and macrophages were separated from other cells using Ficoll-Hypaque (Sigma, St. Louis, MO) gradient separation and selection by adherence to tissue culture plastic. Cells were grown in RPMI containing 5% human serum (Invitrogen) for 24 hr then changed to RPMI containing 5% FBS until differentiation. Differentiation was performed via treatment with 20 ng/ml of IFNγ (Peprotech, Rocky Hill, NJ) and Lipopolysaccharide (Sigma) for 5 days.

### Immunohistochemistry and immunocytofluorescence

Immunohistochemistry was performed with appropriate controls as described [21]. Briefly, five-micron-thick sections of formalin fixed, paraffin embedded tissue or tissue arrays (U.S. Biomax Inc., Rockville, MD; arrays BR2085a and BR805) were de-paraffinized in xylenes, rehydrated, subjected to antigen retrieval using citrate buffer (DAKO, Carpinteria, CA), and staining was performed using the Vectastain Elite ABC System (Vector Laboratories, Burlingame, CA) according to manufacturer’s instructions. Color was developed with diaminobenzidine peroxidase substrate kit (Vector Laboratories) and sections were counterstained with hematoxylin (Sigma Aldrich, St. Louis, MO). The commercially available hornerin antibody was purchased from Novus Biologicals (Littleton, CO) and used as recommended (dilution 1:100). Imaging was performed on an Olympus IX51 microscope (Olympus, Center Valley, PA) and quantified using NIH Image J64 software (threshold standardized; measurement determined as percent area: red). A total of 95 invasive lobular carcinoma and 124 invasive ductal carcinomas and their associated TNM status were analyzed.

For immunocytofluorescence, cells were grown on 8-well chamber slides (Research Products International, Mt. Prospect, IL,) and fixed/permeabilized in ice-cold acetone. Following fixation, cells were blocked in 1% BSA and 5% normal goat serum PBS solution, stained with the indicated primary antibody overnight at 4°C (1:100 dilution), washed and then incubated for 1 hour with an anti-rabbit Alexa Fluor 488 secondary antibody (1:1000 dilution, Invitrogen). DAPI was used to stain DNA. Imaging was performed using the Carol Zeiss LSM510 confocal imaging system (Carl Zeiss MicroImaging, Thornwood, NY) at 63X magnification. For co-localization of hornerin and macrophage specific markers, paraffin embedded human and murine mammary tissue was de-paraffinized, rehydrated, subjected to antigen retrieval as stated above, blocked in a PBS containing 5.0% BSA solution followed by co-incubation of the hornerin antibody (Novus, dilution 1:100) with either F4/80 (Santa Cruz, dilution 1:100; to identify murine macrophages) or CD68 (Novus Biologicals, dilution 1:50; to identify human macrophages) antibodies, washed, and then stained with the appropriate secondary antibodies (1:1000 dilution). Coverslips were applied with VECTASHIELD mounting medium with DAPI (Vector Laboratories). Fluorescent imaging was performed using the Carol Zeiss LSM510 confocal imaging system (Carl Zeiss MicroImaging, Thornwood, NY) at 63X magnification. For quantitation of macrophages in murine mammary tissue, the cells positively stained for hornerin expression and F4/80 were counted in three separate 40x fields; a minimum of three glands per developmental stage was counted.

### Exosome isolation and transmission electron microscopy

For all exosome isolation experiments, cells were grown for at least one passage in growth media that was previously depleted of contaminating microvesicles by overnight centrifugation at 100,000x*g*. Exosomes were isolated as previously described [22,23]. Briefly, supernatants were subjected to a 300x*g* (10 min) followed by 2,000x*g* (10 min) and a 10,000 x*g* (30 min) centrifugation at 4°C to remove cell debris. The supernatants were then centrifuged at 100,000x*g* for 80 min at 4°C three times, with 1X PBS washes in-between each centrifugation. The pelleted exosomes were then solubilized in SDS-sample buffer for western blot analysis.

#### Electron microscopy of purified exosomes

Purified exosomes were centrifuged and fixed in buffered 2.0% glutaraldehyde (Tousimis, Rockville, MD). The pellet was post-fixed in 1.0% osmium tetroxide in cacodylate buffer (0.1 M, pH7.2; Electron Microscope Sciences, Fort Washington, PA) for 1 hour in a room temperature. The pellet was washed in the same buffer, then in acetate buffer (0.1 M, pH4.2) and stained in uranyl acetate (0.5% w/v in acetate buffer) for 1 hour. The pellet was washed in acetate buffer and dehydrated in a series of ethanol (e.g., 35%, 50%, 75%, 95%, 100%) followed by 100% propylene oxide. The pellet was infiltrated in an equal volume of Embed-812 epoxy resin (Electron Microscope Sciences) and 100% propylene oxide overnight at room temperature. The pellet was embedded in a fresh resin and cured at 55°C for 48 hours. Thin sections (80 to 90 nm) were made and mounted on a naked copper grid and stained in uranyl acetate and lead citrate. The sections were examined by electron microscopy (Hitachi 7600, Tokyo, Japan) operated at 80 kV and the images captured by a digital camera (Advanced Microscopy Techniques, Chazy, NY). For TEM analysis, the high-speed pellet was prepared as previously described [24,25], examined and imaged by Hitachi 7600 microscope operated at 80 kV.

### Western blot analysis

Equal concentrations of protein, as determined by the Coomassie Plus Protein Assay (Thermo Scientific, Rockford, IL), were separated by SDS-PAGE under reducing conditions. Membranes were blocked in 5% non-fat milk in TBS buffer with 0.1% Tween (TBST) for 1 hour at room temperature, then incubated with primary antibody (1:1000 dilution for all antibodies; caspase-3, Cell Signaling Technology, Boston, MA; hornerin, Novus Biologicals, Littleton, CO) overnight at 4°C in TBST + 5.0% BSA, washed, and incubated with the appropriate secondary antibody conjugated to horseradish peroxidase (GE Healthcare, Piscataway, NJ) in TBST with 5% milk for 1 hour at room temperature. Peroxidase activity was detected using the enhanced chemiluminescence detection system (ECL Plus, GE Healthcare) as directed. α-Tubulin was used as a control to show equal loading (Santa Cruz Biotechnology, Santa Cruz, CA). Western blots were quantified using NIH ImageJ 64.

### Quantitative real time PCR

Total RNA was isolated using the Qiagen RNeasy kit according to the manufacturer’s instructions (Valencia, CA). RNA was reverse transcribed using MMLV reverse transcriptase (Invitrogen) and primed with oligo-dT and random hexamers (Invitrogen). The cDNA was subjected to RT-PCR amplification using gene specific primers and 2x Brilliant II Sybr Green QPCR Mastermix (Stratagene; La Jolla, CA). Primer sequences are given in Table 1. Quantitative RT-PCR was analyzed via the ^ΔΔ^CT method, and PCR products were visualized by agarose gel electrophoresis.

**Table 1:** Primer Sequences

**Primer**	**Species**	**Sequence (5′- 3′)**	**TM (°C)**
Hornerin	Human	Forward: TTCGTCTTCCAGCTATGGTCAGCA	60
		Reverse: AGTAACTTGAGCCAGACCCGTGTT	
Hornerin	Murine	Forward: TCTCAACGGTTTGGATCTGGCTCA	60
		Reverse: TGTTGACTGCCTTCTGTCTGTCCA	
GAPDH	Human/Murine	Forward: CCCTCCATTGACCTCAACTAC	60
		Reverse: CCACCTTCTTGATGTCATCAT	

### Antibody production

Hornerin N-terminus and C-terminus antibodies were produced by PRIMM (IMGEN Technologies, Cambridge, MA) via immunization of rabbits with a recombinant His-tagged protein (residues N-terminus 14–28 and 199–210, C-terminus 2813 – 2823 and 2834–2846, GI: 388697) and affinity column purified by the Antibody Production and Purification Unit (APPU; National Cancer Institute, Bethesda, MD). Initial affinity column purification was followed by an additional purification using a GE Superdex 200 2.6/60 on an Akta Purifier (GE Healthcare) in PBS containing 0.1% sodium azide. The resulting antibody was validated by western blot analysis against the immunizing protein.

### Statistical analysis

Data was evaluated for significance via t-tests or one-way analysis of variance (ANOVA) with the appropriate *post hoc* analysis (Tukey/Bonferroni) using GraphPad InStat Software version 3.0b (San Diego, CA). Data was considered significant at *P* < 0.05.

## Results

### Expression and localization of hornerin in breast tissue, mammary cells, and exosomes

**Figure 1:**
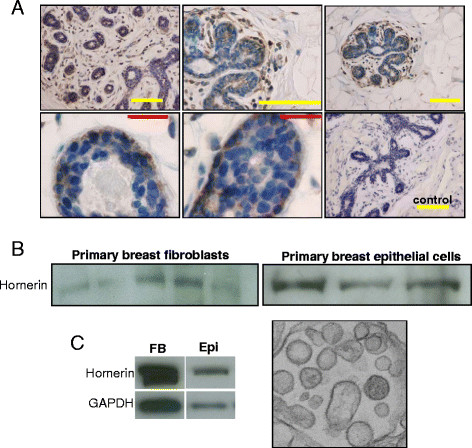
**Immunolocalization of hornerin expression in breast tissue.** (**A**) Reduction mammoplasty tissue sections were subjected to immunohistochemical analysis using a hornerin specific antibody or corresponding negative control. A minimum of 10 patient samples were analyzed. Yellow bar = 200 μM, red bar = 50 μM. (**B**) Western blot analysis of hornerin expression in primary breast fibroblast and epithelial cell whole cell lysates isolated from reduction mammoplasty tissue samples. A minimum of five patient samples were analyzed per cell type. Representative data from five breast fibroblasts and three breast epithelial samples are shown. (**C**) Western blot analysis of exosomes isolated from primary breast fibroblast and epithelial cell cultures and representative TEM image of purified exosomes 30,000X. FB = fibroblasts, Epi = epithelial cells.

Proteomic analysis of the extracellular matrix of normal breast tissue revealed the presence of the S100 family member hornerin [11]. Recent reports have highlighted the importance of the S100 proteins in breast cancer; therefore we further examined the role of hornerin in both normal and cancerous breast tissue. To confirm the presence and localization of hornerin, immunohistochemistry was performed on breast tissue histosections. Hornerin was easily detectable in both the stroma and epithelium, while adipose had significantly lower detectable levels (Figure 1A). There appeared to be a higher concentration of hornerin in the basal and myoepithelial cells compared to the luminal epithelium. In addition to the immunohistochemical analysis, we performed western blot analysis on primary breast fibroblasts and epithelial cells isolated from breast tissue. Hornerin expression was found in both cell types (Figure 1B). Lastly, as hornerin has been reported to be excreted into serum [26], cerebral spinal fluid [27], and in plasma-derived exosomes [28], we examined exosomes isolated from primary breast fibroblast and epithelial cell cultures. Hornerin was readily detectable in the exosome isolations from both cell types (Figure 1C). GAPDH was used as a marker for exosomes and transmission electron microscopy images were used to verify successful exosome isolation [22,23,29].

### Hornerin expression during developmental stages of the murine mammary gland

**Figure 2:**
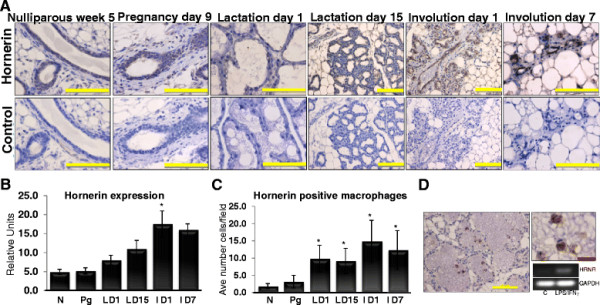
**Hornerin expression throughout the stages of mammary development.** (**A**) Representative images of mammary glands isolated from the indicated stages of development that were subjected to immunohistochemical analysis using a hornerin-specific antibody or corresponding negative control. (**B**) Data represent the mean +/− SD of a minimum of three glands quantitated using Image J64. *P < 0.05 compared to nulliparous and pregnant glands. (**C**) Data represent the mean +/− SD of number of hornerin positive macrophages counted per 40x field; a minimum of three glands was counted at each developmental stage and three fields were counted per slide. * *P < 0.05 compared to nulliparous glands. (**D**) Representative images of immune cells in mammary glands that were subjected to immunohistochemical analysis using a hornerin-specific antibody and the corresponding PCR analysis of hornerin in human monocytes/macrophages isolated from peripheral blood; cells were treated +/− 20 ng/ml of LPS/IFNγ for five days. N = nulliparous week 5, Pg = pregnancy day 9, L D1 = lactation day 1, L D15 = lactation day 15, I D1 = involution day 1, I D7 = involution day 7. Yellow bar = 200 μm, Red bar = 50 μm.

The previously reported distinct regulation of hornerin expression during epidermal cell differentiation prompted us to observe its regulation throughout postnatal mammary gland development. Abdominal mammary glands isolated from both FVB and Balb/c mice were obtained from each of the significant developmental stages and subjected to immunohistochemical analysis using a hornerin specific antibody. Hornerin expression temporally increased during the differentiation of the gland throughout pregnancy, with maximal expression observed during lactation and involution (Figure 2A, B). Quantitation of the staining demonstrated a significant increase in expression during the early stages of involution (*P <* 0.05, representative images of quantitation method shown in Additional file 1: Figure S1). During involution, the changes occurring in the gland include the reabsorption of residual milk, loss of the epithelium by apoptosis, clearance of dying cells, and regrowth of the epithelial and stromal cells [30]. We also observed a significant increase in hornerin staining within the macrophages specifically during lactation and involution compared to nulliparous tissue (*P <* 0.05; Figure 2C, D and Additional file 2: Figure S2). To complement the macrophage staining observed in the murine tissue, human peripheral blood monocytes were isolated and treated in the presence or absence of LPS/INFγ (20 ng/ml), the RNA was isolated and transcript abundance was measured via PCR. Low levels of hornerin were present in the undifferentiated cells, while treatment with LPS/INFγ stimulated a significant increase in hornerin expression in the macrophages. The observed increase in hornerin expression in the differentiated macrophages compared to undifferentiated monocytes suggests the possibility a functional role for hornerin in phagocytic macrophages.

### Hornerin expression in breast cancer

**Figure 3:**
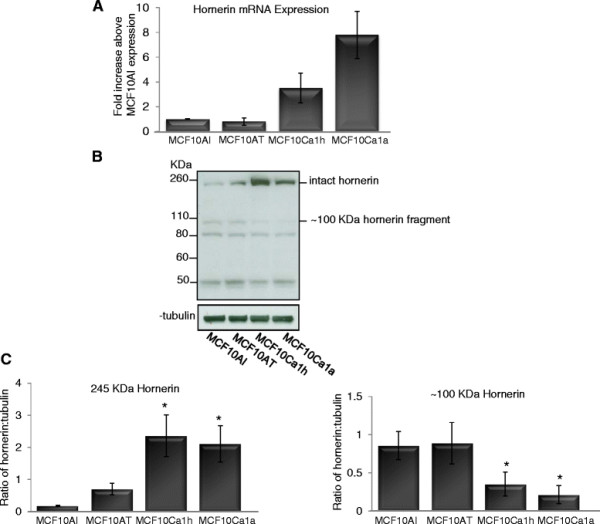
**Hornerin expression in an *in vitro *model of breast cancer progression.** (**A**) Quantitative real time PCR analysis of hornerin transcript abundance in proliferating MCF10A cell line series. Quantified data were normalized to the housekeeping gene GAPDH. (**B**) Western blot analysis of hornerin expression in proliferating MCF10A cell line series; α-tubulin was used as a loading control. (**C**) Data represent mean +/− SD of three independent experiments. Quantified values were expressed relative to corresponding abundance of tubulin protein expression *P < 0.05.

Given the emerging role of S100 proteins in breast cancer, we investigated hornerin expression in an *in vitro* breast cancer progression model. MCF10A cells are a spontaneously immortalized breast epithelial cell line that have been extensively used to study normal breast epithelial function [31]. Through transfection of the parental line with a constitutively active H-Ras, and subsequent selection for increasingly aggressive tumor formation from cells recovered from xenograft tumors, the pre-malignant MCF10AT, malignant MCF10Ca1a, and metastatic MCF10Ca1h cell lines were developed [20,32-34]. This series of cell lines provides a unique opportunity to study breast cancer progression, induced in a defined method, in a common cell background. Quantitative real-time PCR was used to determine the expression levels of hornerin in each stage of the MCF10A cancer progression model. Transcript abundance exhibited a trend to increase as the tumorigenicity of the cells progressed (Figure 3A). Western analysis confirmed the presence of hornerin in the cells, and a similar pattern of increased expression was observed in the more tumorigenic cell lines. At the protein level, the MCF10Ca1a and MCF10Ca1h cell lines had significantly more protein compared to the normal and premalignant cell lines (*P <* 0.05). The transcriptional characteristics of MCF10A cells share many features of basal progenitor cells suggesting that these cells may represent a multipotent lineage [35]. Our data localizing the expression of hornerin in the basal/myoepithelial cells of the human breast (Figure 1) is consistent with the expression of hornerin in the MCF10A cell line.

Western analysis also demonstrated posttranslational proteolytic processing of hornerin, similar to previous studies in skin [12-15]. Fragments at 50, 80, and 100 kDa were observed. However, only the 100 kDa fragment showed differential regulation when comparing the premalignant to the malignant cell lines (Figure 3B, C; *P <* 0.05).

**Figure 4:**
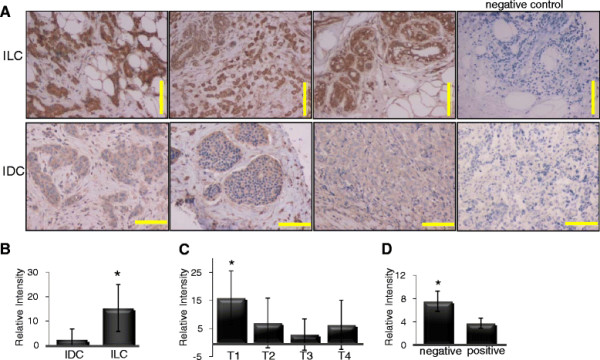
**Correlation of hornerin expression with breast cancer subtype and TNM staging.** (**A**) Representative images of breast cancer biopsies that were subjected to immunohistochemical analysis using a hornerin-specific antibody or corresponding negative control (219 individual patient samples analyzed). Yellow bar = 200 μM. Hornerin expression in correlation with (**B**) breast cancer subtype IDC = invasive ductal carcinoma. ILC = invasive lobular carcinoma, (**C**) tumor grade, and (**D**) lymph node metastasis. Data represent mean relative intensity +/− SD. *P < 0.05.

The presence of hornerin in each stage of the MCF10A breast cancer progression model prompted us to investigate hornerin expression in correlation to breast cancer subtype. Breast cancer tissue arrays were analyzed for intensity of hornerin expression via immunohistochemistry. A total of 125 invasive ductal carcinomas (IDC) and 95 invasive lobular carcinomas (ILC) were analyzed and results showed a significant increase in hornerin expression in the ILC (Figure 4A, B, *P <* 0.05). A significant correlation was also found with increased hornerin expression and favorable TNM grade (Figure 4C, *P <* 0.05). Hornerin expression was increased in tumors that invaded the submucosa (T1) compared to more invasive tumors (T2 - T4) as well as in tumors that lacked pathological lymph node involvement (Figure 4D*P <* 0.05). No correlation was found with hornerin expression and patient age.

### Subcellular localization of hornerin fragments

**Figure 5:**
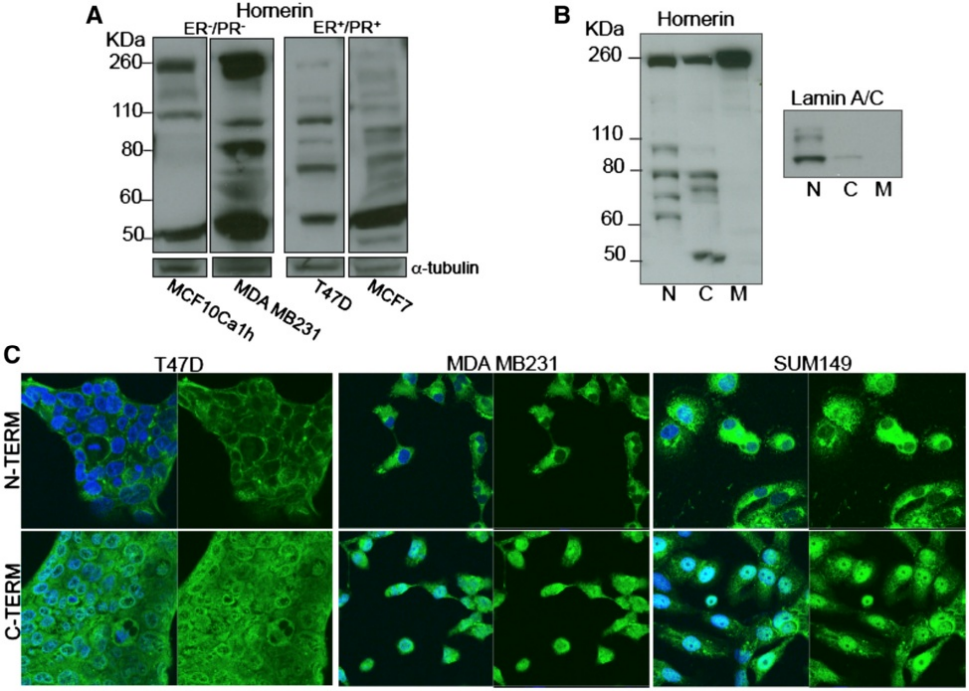
**Localization of hornerin fragments in breast cancer cells.** (**A**) Representative western blot of hornerin products in breast cancer cell lines. ER = estrogen receptor, PR = progesterone receptor; α-tubulin was used as a loading control. (**B**) Representative western blot of MCF10AI subcellular fractionated products. N = nuclear, C = cytoplasmic, M = membrane fraction. Lamin A/C indicates purity of subcellular fractions. (**C**) Confocal microscopy images of hornerin localization using antibodies directed against the proteins N-terminus and C-terminus in the indicated breast cancer cell lines (63x magnification). For each cell line, the top panel shows hornerin staining (green), while the bottom panel shows both the nuclear (DNA stained with DAPI, blue) and hornerin staining (green).

In addition to the breast cancer biopsies, the transcript abundance of hornerin was investigated in a panel of breast cancer cell lines. No correlation was observed with estrogen receptor (ER) and progesterone receptor (PR) status and hornerin expression (MCF7, T47D, ZR75.1, MCF10AI, MCF10Ca1h, MDA MB231, SUM159, MDA MB468 cell lines; analyzed via PCR, *data not shown* as well as referenced in [35]). However, western blot analysis using a commercially available antibody directed against the N-terminus of the protein indicated a potential difference in fragmentation of hornerin between the ER/PR negative and positive cell lines. The ER/PR positive cells appeared to have overall lower levels of the intact 245 KDa hornerin compared to the ER/PR negative cell lines (Figure 5A). To further investigate the function of protein fragmentation, the localization of the fragments within subcellular compartments was investigated. The MCF10AI cells had relatively even levels of hornerin fragmentation (Figure 3) and were therefore used for subcellular fractionation experiments. The nuclear fraction contained a higher level of the smaller hornerin fragments, while the membrane fraction contained primarily the intact protein (Figure 5B). To visualize the localization of the fragment *in situ*, antibodies directed against both the N-terminus and C-terminus of the protein were developed. While the C-terminus antibodies were not effective in western blot analysis, all antibodies developed were successful for immunofluorescence. Confocal microscopy demonstrated a pattern of predominantly cytoplasmic/membrane localization of hornerin using the N-terminus antibody, with comparatively low levels of nuclear localization while the C-terminus antibody demonstrated a comparably stronger nuclear accumulation for all cell lines tested (Figure 5C).

### Cell death induces hornerin expression and increased fragmentation

**Figure 6:**
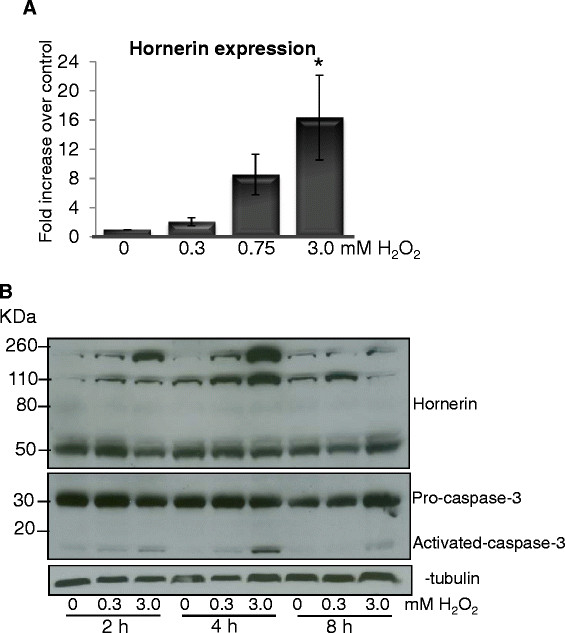
**Induction of cell death events increases hornerin expression and fragmentation.** (**A**) Quantitative real time PCR analysis of hornerin transcript abundance in MCF10AI cells treated overnight with increasing concentrations of H2O2. Data are the mean +/− SEM of three independent experiments; * *P <* 0.05. (**B**) Representative western blot of hornerin fragments, caspase 3 and cleaved caspase 3 in MCF10AI cells treated with the indicated concentrations of H2O2. α-Tubulin was used as a loading control.

Previous studies show that S100 proteins generate reactive oxygen species (ROS) and are strong inducers of apoptosis [36,37]. Attempts to produce recombinant hornerin in both bacteria and insect cells failed due to the exceptionally large size and repetitive sequence of the protein, therefore, we analyzed changes in endogenous hornerin expression in response to ROS and cell death inducing events. MCF10AI cells were treated overnight with increasing amounts of H_2_O_2_ and transcript abundance was measured via quantitative PCR. Hornerin expression was increased in a dose-dependent manner with a significant increase in expression observed with 3.0 mM treatment (*P <* 0.05, Figure 6A). A similar increase in hornerin expression was observed in both T47D and MDA MB231 cells, as well as with 5-fluorouracil treatment in the MCF10AI cells (Additional file 3: Figure S3). These data suggest a common mechanism of increased hornerin expression in response to apoptosis/cell necrosis inducing events. Western blot analysis showed extensive degradation/fragmentation with H_2_O_2_ treatment after overnight treatment; therefore a more extensive timecourse and dose response were performed (Figure 6B). As early as two hours post-treatment an increase in the ~100 KDa fragment of hornerin was evident with the higher dose of H_2_O_2_. The increase in fragmentation was maximal at four hours post-treatment. A similar pattern of capase-3 activation was observed, suggesting a correlation between hornerin fragmentation and apoptosis. Overall these data support a role for hornerin in cell death related events, similar to other S100 proteins.

## Discussion

The mammary gland is proposed to have developed during evolution from the transformation of an apocrine sweat gland [38], and it is well documented that during embryonic development the mammary gland arises from local thickening of the ventral embryonic epidermis [16]. Together these two observations support the potential role of hornerin, a protein involved in the cornification of skin, in mammary cell function. Herein, we demonstrate hornerin expression and localization in breast tissue and breast cancer, as well as changes in regulation during cell apoptotic/necrotic events. These data highlight the multifunctional role of hornerin in tissue additional to skin.

Lower levels of H_2_O_2_ stimulate apoptosis, while high levels induce necrosis in breast cancer cells [39]. Our data show that both low and high concentrations of H_2_O_2_ induced hornerin expression and fragmentation in all cell lines observed, corresponding with activation of caspase-3. The immense size, complex and repetitive sequence of hornerin resulted in failed attempts to successfully produce a recombinant protein, overexpress the protein, or significantly knock down the levels in cell lines; therefore, we were unable to directly determine whether the increase in mRNA levels and protein fragmentation are promoting cell survival or promoting cell death. However, other S100 protein family members have been shown to promote both cell survival and apoptosis in mammary epithelial cells and breast cancer [5,40]. S100A7, S100A8 and S100A9 are direct downstream targets of, and transcriptionally repressed by, BRCA1 [41]. Bioinformatic analysis using PHOSIDA posttranslational modification database predicts three prominent proteins implicated in the control of cellular response to DNA damage as transcription factors for hornerin (DNA damage response kinase, casein kinase 1 (CK1) and glycogen synthase kinase-3 (GSK-3)). Furthermore, a direct interaction of hornerin with PinX1 was identified in a large scale mapping of protein-protein interactions by mass spectrometry [42]. PinX1 has been identified as a major tumor suppressor, essential for telomerase activity and maintaining chromosome integrity [43]. These observations along with our data demonstrating a significant upregulated of hornerin during early stages of involution and that hornerin expression is upregulated in less aggressive, lymph node negative, T1 breast tumors (Figure 4), strongly suggests a role for hornerin in promoting apoptosis and tumor suppression.

Hornerin was detected in exosomes secreted from mammary cells. As hornerin was localized to the cell membrane of mammary cells (Figure 5) hornerin may have been incorporated into the exosomes during the fusion of the multivesicular bodies with the plasma membrane and subsequent release from the cell, or as a component deposited within the exosome. Cells release exosomes for multiple purposes, including the eradication of obsolete proteins and as a mode of intracellular communication [44]. The latter is especially true for immune cells, and it is of note that we observed significant hornerin expression in stromal immune cells during lactation and involution. Family members S100A8 and S100A9 are excreted into the extracellular environment via a protein kinase C dependent mechanism in neutrophils, monocytes and myeloid progenitors in response to cell damage and act as danger signals that activate other immune cells and endothelial cells [45].

Most of the S100 genes, including hornerin, are clustered on chromosome region 1q21, a region frequently altered in epithelial tumors [45]. Our data show an increase of hornerin expression in invasive luminal breast cancer patient samples compared to invasive ductal carcinomas, and had significant correlation with tumors of a less aggressive pathology. Additionally, the ER/PR positive cell lines displayed more hornerin fragmentation than the more aggressive ER/PR negative cell lines. It is possible that the fragmentation of hornerin is an important mechanistic step in controlling hornerin action similar to that previously demonstrated in prostate cancer cells [46] or the MCF7 breast cancer cell line [47]. It is of note that the MCF10A breast cancer cell line progression model showed increasing amounts of hornerin expression as the tumorigenicity of the cells progressed. This observation is in contrast to the data observed in the breast cancer tissue array (i.e. the less aggressive tumor tissue had higher levels of hornerin expression). We hypothesize that the fragmentation and localization of the fragments relates to the function of hornerin, thereby explaining these discrepancies. Indeed, less hornerin fragmentation is observed in the more aggressive MCF10A lines, similar to less fragmentation observed in the ER/PR negative breast cancer cell lines (Figure 5), which are inherently more invasive and tumorigenic compared to the ER/PR positive cell lines [35,46]. It is possible that the enhanced fragmentation directly relates to the increase in intensity and abundance of hornerin detected in the lobular breast tissue tumor samples compared to the ductal carcinomas.

Our scanning confocal microscopy data show differential localization of hornerin. Specifically, using antibodies directed against the N-terminus of hornerin we showed enhanced membrane staining and minimal nuclear localization, while nuclear localization was robustly evident when using an antibody directed against the C-terminus. This differential subcellular localization of the fragments may suggest alternative functions for full length and fragmented hornerin. A similar mechanism of subcellular localization of protein fragments was recently described for Ral binding protein-1 (RalBP1), a protein implicated in Ras function [47], and other multiple functions localized to various subcellular regions including the nucleus, the actin cytoskeleton, and with the mitotic spindle and centrosome during mitosis [47]. Moreover, RalBP1 was shown to be proteolyzed into several fragments with differential subcellular localization, suggesting the proteolyzed fragments mediated different cellular functions.

Furthermore, differential subcellular localization of other S100 family members has been reported [48]. Distinct intracellular localization of S100A6 and S100A4 was shown to be dependent on calcium concentrations in the MDA-MB231 metastatic epithelial breast adenocarcinoma cells and cervical carcinoma HeLa cells [48]. The authors propose the S100 proteins are involved in tumor cell calcium homeostasis. Additionally, S100A11 was shown to change from a strictly nuclear localization in normal breast tissue to a more cytoplasmic localization in breast tumors. The authors hypothesized that in normal breast S100A11 translocates to the nucleus which increases transcription of negative regulators of cell growth, while in cancer the loss of nuclear translocation may lead to an inability to control cell growth [49].

## Conclusion

Our data highlight the potential role for hornerin, an S100 protein family member, in mammary cell function. We demonstrate hornerin expression in breast epithelial cells, stromal fibroblasts and macrophages, and show unique regulation of hornerin expression during distinct phases of mammary development. Furthermore, we show a decrease in hornerin expression in invasive ductal carcinomas compared to invasive lobular carcinomas and less aggressive breast carcinoma phenotypes, and altered cellular expression of hornerin during induction of apoptosis. Finally, we demonstrate the presence of post-translational fragments that display differential subcellular localization, which opens new possibilities for the existence of fragment-specific functions of hornerin.

## Competing interests

The authors declare that they have no competing interests.

## Authors’ contributions

JMF and BKV conceived the project. JMF designed and performed all experiments and wrote the manuscript. SDO assisted in experiments. EG edited the manuscript and assisted in experiments. PG designed and produced the antibodies. All authors contributed to the analysis of data and approval of the final manuscript.

## Supplementary Material

Additional file 1 Figure S1. Representative images from the quantitation of hornerin expression in murine mammary tissue. Click here for file

Additional file 2 Figure S2. Co-localization of hornerin and macrophage expression in human breast and murine mammary tissue. Click here for file

Additional file 3 Figure S3. Regulation of Hornerin expression. Click here for file
