# Supplementary figures and images for: Hornerin, an S100 family protein, is functional in breast cells and aberrantly expressed in breast cancer
Source: BMC Cancer. 2012 Jun 22;12:266. doi: 10.1186/1471-2407-12-266 (PMC3464886; doi:10.1186/1471-2407-12-266)

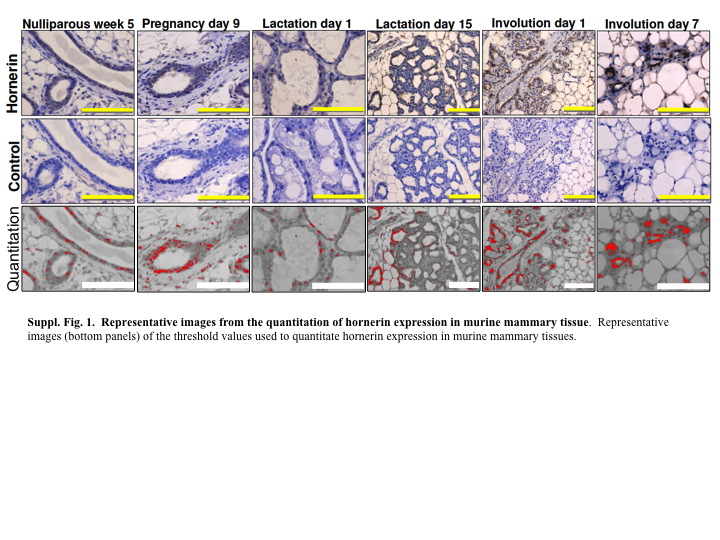

Supplement: Additional file 1 — Figure S1. Representative images from the quantitation of hornerin expression in murine mammary tissue. [file 1471-2407-12-266-S1.tiff]

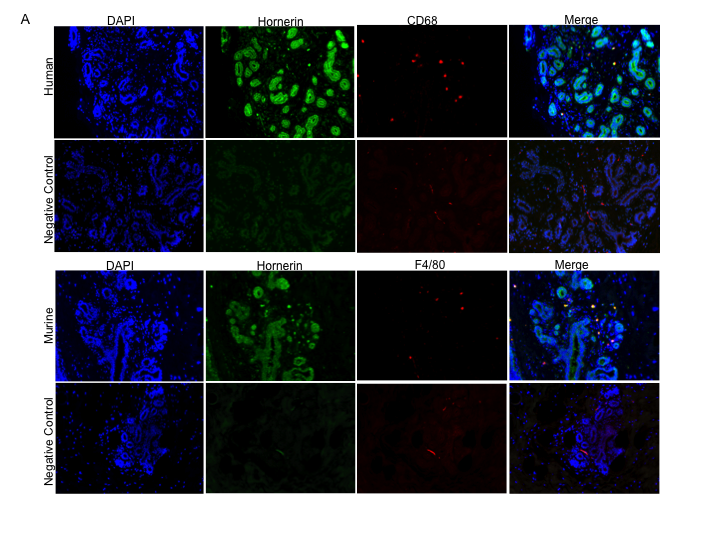

Supplement: Additional file 2 — Figure S2. Co-localization of hornerin and macrophage expression in human breast and murine mammary tissue. [file 1471-2407-12-266-S2.tiff]

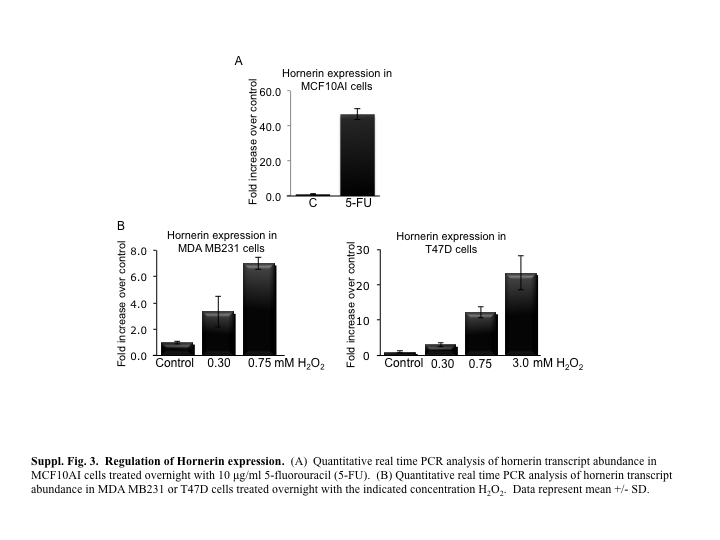

Supplement: Additional file 3 — Figure S3. Regulation of Hornerin expression. [file 1471-2407-12-266-S3.tiff]
